# Otoacoustic Emissions in Children with Long-Term Middle Ear Disease

**DOI:** 10.3390/life10110287

**Published:** 2020-11-18

**Authors:** Milaine Dominici Sanfins, Luisa Frata Bertazolli, Piotr H. Skarzynski, Magdalena Beata Skarzynska, Caroline Donadon, Maria Francisca Colella-Santos

**Affiliations:** 1 Child and Adolescent Heath Program, Faculty of Medical Sciences, State University of Campinas, Campinas, São Paulo 04515-030, Brazil; c101841@dac.unicamp.br; 2 Advanced Electrophysiology and Neuroaudiology Center, Department of Electrophysiology, São Paulo 04515-030, Brazil; 3 Faculty of Medical Sciences, State University of Campinas, Campinas, São Paulo 04515-030, Brazil; lubertazolli@gmail.com; 4 Institute of Physiology and Pathology of Hearing, 00-002 Warsaw, Poland; p.skarzynski@inz.waw.pl; 5 Department of Heart Failure and Cardiac Rehabilitation, Faculty of Medicine, Medical University of Warsaw, 00-002 Warsaw, Poland; 6 Institute of Sensory Organs, 00-002 Warsaw, Poland; m.skarzynska@csim.pl; 7 Institute of Physiology and Pathology of Hearing, World Hearing Center, 05-830 Kajetany, Poland; 8 Center of Hearing and Speech, 05-830 Kajetany, Poland; 9 Department of Human Development and Rehabilitation (DHDR), Faculty of Medical Sciences, State University of Campinas, Campinas, São Paulo 04515-030, Brazil; mfcolella@fcm.unicamp.br

**Keywords:** otitis media, children, otoacoustic emission, hearing, ventilation tubes

## Abstract

Introduction: Otoacoustic emissions (OAEs) evaluate the functional status of the cochlea. Repeated otitis media (OM) can cause changes in the peripheral structures of the auditory system, and, in this way, middle ear infection may irreversibly damage the middle ear, or even the cochlea. Objectives: To analyze the results of transiently evoked otoacoustic emissions (TEOAEs) and distortion product otoacoustic emissions (DPOAEs) in individuals with a history of OM. Method: Participants with 8 to 16 years of schooling were split into two groups: a control group (CG) of 50 subjects who had no history of otological disease and an experimental group (EG) of 50 subjects who had a history of recurrent otitis in childhood and had consequently undergone myringotomy to insert bilateral ventilation tubes. All children underwent basic audiological assessment (tonal audiometry, speech audiometry, and immittance testing) and otoacoustic emission testing (TEOAEs and DPOAEs). Results: There were no significant differences between the groups when audiometrically tested via air and bone conduction. OAEs were found in all CG subjects. For the EG, there were no TEOAE responses in 17 ears and no DPOAEs in nine ears; response amplitudes were lower at all frequencies. The emission level and the signal-to-noise ratio were statistically different between the two groups, and OAEs in the EG were statistically smaller compared to the GC. Conclusion: In the EG, responses were more likely to be absent and were of statistically smaller amplitude compared to the CG. A history of repeated OM apparently interferes with the generation and transmission of TEOAEs and DPOAEs.

## 1. Introduction

Otitis media with effusion (OME) is one of the most common childhood diseases and can affect about 2/3 of children in the first 5 years of life [1]. The high incidence of OME in children can be explained by the immaturity of the immune system and by the structural and functional immaturity of the Eustachian tube [2,3]. OME is an inflammation of the middle ear with the presence of serous or mucous secretion, an intact tympanic membrane, but with no clinical manifestations of acute infection [4]. The secretions in the middle ear interfere with the transmission of sound through the ossicle/tympanic system, often leading to mild to moderate conductive hearing loss [5,6,7,8].

There are different ways of treating OME episodes. They can be conservative, such as insufflation of the Eustachian tube together with decongestant medication or, in cases where these are not effective, one can opt for surgical means, especially in cases of recurrent and lasting OME [4,9]. To treat the condition, placement of a ventilation tube after myringotomy is the most commonly used surgical procedure in children [4]. Myringotomy allows fluid accumulated in the middle ear to drain, aerates the middle ear, and thereby restores hearing; in this way, it can minimize the effects of OME on language development [10].

While the literature typically associates OME with conductive hearing loss, other research has shown that the basal turn of the cochlea can also be involved [11].

In terms of the cochlea, otoacoustic emissions (OAEs) are sounds generated by the activity of outer hair cells, and their presence over the auditory frequency band indicates normal or near-normal cochlear functioning [12]. The sounds generated in the cochlea in response to a stimulus are transmitted by the middle ear to the external acoustic meatus, where they can be picked up by a probe. The presence of otoacoustic emissions indicates that the pre-neural reception mechanism in the cochlea is able to respond to sound; it also indicates that the middle ear mechanism is intact and can allow sound to be transmitted through the tympano-ossicle system to the external acoustic meatus [12,13]. Thus, impairments of the middle ear and cochlea have a considerable effect on the response level of OAEs [14], making OAEs a highly sensitive test for cochlear changes or minor middle ear dysfunction [15,16]. In addition, OAEs are considered an effective method for detecting auditory alterations at an early stage, since they can pick up changes before pure tone audiometry is able to [13]. OAEs cannot take the place of tympanometry, but they can be a good complementary way to screen for OME in children [17]. Both transient otoacoustic emissions (TEOAEs) and distortion product otoacoustic emissions (DPOAEs) are considered important for the early diagnosis of noise-induced hearing loss [18,19]. Researchers have pointed to decreased DPOAE amplitude due to compromised outer hair cells as a possible cause of hidden hearing loss; moreover, DPOAEs are less affected by natural variations in pressure, compliance, or compensation [20,21].

Several studies have shown that episodes of childhood OME have significant long-term impacts on the peripheral and central auditory system [4,22,23,24]. In the peripheral system, lesions have been found in the outer hair cells at the base of the cochlea, even when psychoacoustic thresholds were within the normal range. According to Norowitz et al. (2019), an abnormal OAE response can be detected years after OM has resolved. In other words, this disease may have a longer lasting effect on otological and auditory status than previously thought [25]. Thus, in children who have had long term middle ear disease it can be hypothesized that OAEs can appear at reduced amplitude at particular frequencies or even be completely absent.

The aim of the present study was to analyze the long-term effects of OME on the peripheral auditory system in children who have had a history of bilateral OME by measuring their TEOAE and DPOAE responses.

## 2. Materials and Methods

### 2.1. Statement of Ethics

This study was approved by the Research Ethics Committee of the State University of Campinas, Unicamp (protocol number 889074) and by the São Paulo State Research Support Foundation (protocol number 04039-1). Data were collected between October 2013 and January 2016 at the Laboratory of Audiology at the Department of Human Development and Rehabilitation/School of Medical Sciences of the State University of Campinas. Informed consent for research was obtained from the parents of all participants after an explanation of the nature, purpose, and expected results of the study.

### 2.2. Participants

A total of 100 school children belonging to the elementary section of a public school participated in this study, 57 female and 43 male, aged between 8 and 16 years. The subjects were divided into two groups:

(i) Control group (CG) consisted of 50 students (31 female and 19 male) with no history of OME and no school complaints.;
(ii) Bilateral experimental group (EG) consisted of 50 students (26 female and 24 male) with a documented history of three episodes of OME who had undergone surgery for insertion of bilateral ventilation tubes in the first 6 years of age and who had normal hearing at the time of evaluation.

There was a predominance of females in both groups (62% CG and 52% EG). The mean age of the CG was 10.8 years, with a minimum age of 8 years and maximum of 14 years, while in the EG the mean age was 11.1 years, with a minimum age of 8 years and maximum of 16.

The subjects in the CG were selected by the pedagogical coordinator of the school, who analyzed the children’s school performance through a questionnaire and later by the researcher with regard to ear complaints. The EG was selected by the researcher through an analysis of the medical records of the State Hospital and finding those who, between 2000 and 2009, had undergone surgery for insertion of bilateral ventilation tubes. All selected subjects were invited through telephone contact with those responsible for them.

#### Inclusion Criteria

The inclusion criteria were defined as:

(i) Control group (CG)
  - Air conduction threshold below 20 dB HL for octaves from 0.25 to 8 kHz;
  - Bone conduction thresholds below 15 dB HL for octaves 0.5 to 4 kHz;
  - Normal middle ear function (Type A) defined as a peak compliance within 0.3 to 1.3 mmhos and peak pressure within −100 to +20 daPa with the presence of ipsilateral and contralateral acoustic reflexes bilaterally between 70 and 100 dB for 0.5, 1, 2, 3, and 4 kHz [26];
  - Typical development: good performance at school and good language development; absence of attention, auditory, or otorhinolaryngological disorders.
(ii) Experimental group (EG), in addition to the above criteria, included the following:
  - Type A tympanogram with compliance between 0.3 and 1.3 mmhos and pressure between −100 and +200 daPa;
  - Absence of middle ear infection for a period of 12 months before the date of evaluation;
  - Documented history of three episodes of OME in the first 6 years of life and bilateral myringotomy with one-time ventilation tube insertion.

### 2.3. Procedures

#### 2.3.1. Audiological Evaluation

**a.** The audiometric evaluation involved air conduction thresholds at 0.25, 0.5, 1, 2, 3, 4, 6, and 8 kHz and bone conduction at 0.5, 1, 2, and 4 kHz. The auditory threshold was considered normal up to 15 dB in air conduction and up to 20 dB in bone conduction according to the classification of Davis and Silverman [27]. The evaluation was performed in an acoustic booth with an Interacoustics AC40 audiometer and TDH39 headset calibrated according to ISO-389 and IEC-645 standards.

**b1.** Speech Recognition Threshold: A list of disyllables was adopted, and the final result was the intensity at which the participant scored 50% of the words presented.

**b2.** Speech Recognition Index: The test was performed with a list of monosyllabic words 40 dB above the mean tonal threshold of 0.5, 1, and 2 kHz and was considered normal if the number of correct answers was between 88 and 100%. 

**c.** Immittance audiometry (tympanometry and acoustic reflex): Tympanometry was performed with a 226 Hz tone at 85 dB SPL with pressure swept from −400 to 200 daPa. Ipsilateral and contralateral acoustic reflexes were sought at 0.5, 1, 2, and 4 kHz. Subjects presented peak maximum compliance around 0 daPa and an equivalent volume of 0.3 to 1.3 mL according to the proposal of Jerger (1970) [26]. Immittance audiometry was performed using an Interacoustics 235 h audiometer. All equipment was calibrated according to ISO-389 and IEC-645 standards. All subjects who presented normal answers in the basic audiological evaluation then proceeded to an electrophysiological evaluation of their hearing.

#### 2.3.2. Evoked Otoacoustic Emission Evaluation

The evaluation of transient otoacoustic emissions (TEOAEs) and distortion product otoacoustic emissions (DPOAEs) was done in subjects who presented normal responses to meatoscopy and to the basic audiological tests. OAE evaluation was done with Biologic Navigator Pro equipment (Natus, Pleasanton, CA, USA) using Scout software in an acoustically prepared room. Subjects remained comfortably seated in a reclining chair.

TEOAEs were evoked with a standard click stimulus at around 80 dB pe SPL at 1, 1.5, 2, 3, and 4 kHz and recorded in a standard 20 ms window. Exactly 260 low-noise responses were collected in the nonlinear acquisition mode. The noise rejection level was set to its default value of 47 dB SPL and fitting of the probe was inspected prior to each recording. TEOAEs were considered present if response signal to noise ratio (SNR = otoacoustic amplitude minus noise floor in dB SPL) was ≥ 6 dB with a reproducibility ≥ 70% in at least three frequencies with an overall SNR ≥ 6 dB SPL and a global reproducibility parameter ≥ 50% [28,29].

For DPOAEs, the ear canal response was monitored to check the fitting of the probe. DPOAEs in the form of a DP-gram for f2 = 750, 984, 1500, 2016, 3000, 3984, 6000, and 7969 Hz (L1 = 65 dB SPL and L2 = 55 dB SPL) at the default value for the f2/f1 ratio of 1.22, values that were generally effective for most patients and frequencies. In addition, the data recorded at 65 and 55 dB SPL (L1 and L2, respectively) were used to establish a DP-gram, in which the DPOAE response level was plotted as a function of f2 frequency, at fixed L1 and L2. DPOAE were considered present when the SNR was equal to or greater than 6 dB [30].

## 3. Results

### 3.1. Audiological Evaluation

Table 1 demonstrates that, for TEOAEs and DPOAEs, there were no significant differences between the groups for audiometric frequencies tested via air and bone conduction. In addition, there was no more than a 5 dB HL difference between the means of the thresholds at all frequencies from 0.25 to 8 kHz (air conduction) or between the means of the thresholds from 0.5 to 4 kHz (bone conduction). Moreover, all hearing thresholds were below 15 dB bilaterally for both groups and all children had Type A tympanometric curves.

### 3.2. Transient Evoked Otoacoustic Emissions (TEOAEs)

It was possible to verify the presence of TEOAEs in all CG children, but in the EG there were 17 ears with no TEOAE response (Table 2). Table 3 compares TEOAEs over 1.2–3.4 kHz, and demonstrates that there was no statistical difference between the groups in terms of age or gender. There was a statistically significant difference between levels of the CG and the EG (*p* < 0.001) and in SNR (transient response level minus noise floor) (*p* < 0.001) in both ears. The CG had higher mean and median values, while the EG showed higher standard deviations, with the exception of the transient emission (TE) and noise floor (NF) measurements in the right ear (Table 4).

### 3.3. Distortion Production Otoacoustic Emissions (DPOAEs) 

The presence of DPOAEs was verified in all CG children, but there were nine ears in the EG which had no DPOAE responses (Table 2). Table 5 demonstrates that there was no statistical difference between the groups in terms of age and gender (for DPOAEs between 1818 and 7206 Hz). There was a statistically significant difference in DPOAE levels at all frequencies, with more robust responses in the CG compared to the EG. Statistically significant differences were observed in the noise floor (NF) only for 7206 and 5083 Hz. For DP—NF (distortion product response minus noise floor), statistically significant responses were observed at all frequencies except for 2542 Hz. However, even though there was no statistically significant difference at 2542 Hz, the CG still had a higher level than the EG (Table 6).

## 4. Discussion

Analyzing the mean responses of the CG and EG based on frequencies from 250 Hz to 8 kHz, both groups had equal hearing thresholds; similarly, the tympanometry results showed that both groups had normal middle ear function. Thus, it was found that OME in the EG had not caused a long-term negative effect. It seemed that the middle ear had recovered after the course of the disease. Specifically, the middle ear had probably not interfered with the responses of TEOAEs or DPOAEs. That is, the generation of otoacoustic emissions is related to the effect of otitis media toxins on the outer hair cells, while the transmission of otoacoustic emissions is related to the path taken through the middle ear, which may be altered due to sequelae in the tympanic–ossicular system.

In the present study, there were more females in both the control group and the experimental group. Previous studies performed in children with a history of OME have shown a higher prevalence of pathology in males due to less efficient tubal function in boys [31,32,33].

In terms of audiometric thresholds for both air and bone conduction, there were no significant differences between the groups. There was no more than a 5 dB difference between the means of air conduction thresholds from 0.25 to 8 kHz and between the means of bone conduction thresholds from 0.5 to 4 kHz. These findings are in general agreement with other researchers who have reported no alteration in psychoacoustic threshold or OAE responses [34,35], even though otoacoustic emissions are highly sensitive to outer hair cell changes.

According to Pienkowski [20], OME might be the cause of hidden hearing loss, since even though audiometric measures may remain normal, hidden damage could impair sound localization and alter the processing of auditory information. It is therefore highly recommended that patients with OME who have normal audiograms be monitored electroacoustically and electrophysiologically in order to be sure that the best audiological monitoring and intervention is provided. The high sensitivity of the OAE test probably explains why, in this study, OAE abnormalities could be detected even though other tests, such as psychoacoustic threshold and immittance audiometry, were normal.

One limitation of the present study was the absence of high-frequency audiometry, since OME can, in the long term, cause a deterioration in high-frequency hearing while audiometric thresholds at 0.25 to 8 kHz remain within the normal range [34,36,37,38,39]. High-frequency audiometry can be used to monitor episodes of aggravated OME [40], and can pick up the effects of OME on cochlear function [41].

All the children in the control group had normal TEOAE and DPOAE responses; in contrast, 17 ears in the experimental group had absent TEOAE responses and nine ears had absent DPOAE responses. The diminished responses can be attributed to loss of outer hair cells at the base of the cochlea, perhaps caused by toxic substances permeating from the middle ear to the cochlea through the round window membrane, or by ultra-structural lesions in the inner ear of children with a history of OME [42,43,44]. Our findings corroborate the literature and point to a history of OME interfering with OAE generation. Another study [16] has also reported that a history of OME can disrupt cochlear amplification, for which outer hair cells are essential, suggesting that OME may lead to impairment of these structures.

By comparing TEOAEs between the groups, we saw a statistically significant difference in TE and SNR in both ears, with more robust responses in the mean values of the CG compared to the EG. The EG also showed generally higher standard deviations (with the exception of TE and NF measurements in the right ear). These results show that TEAOEs are highly sensitive to cochlear changes, implying they can be used to monitor children with a history of OME, especially before and after medical treatment or surgical intervention [45]. There are reports of left/right differences in otoacoustic emission amplitudes, even when there is symmetry in the pure tone thresholds in both ears, and this may be related to a difference in how otoacoustic emissions originate and how the inner ear processes pure tones [46]. The present study has observed greater TEOAE amplitudes in the right ear of the CG than in the left; however, in the EC, the results were the opposite, with greater TEOAE amplitudes in the left ear. The greater amplitude in the right ear suggests enhanced auditory processing in the right ear, the so-called right ear advantage. Because of this, children with a history of OME could have a disruption in this pattern which has an effect on all auditory processing.

The absence of emissions in some of the children indicates some kind of damage to the cochlea, although not necessarily the outer hair cells. Because air conduction thresholds, bone conduction thresholds, and immittance audiometry were normal in these children, we can rule out middle ear problems. OAE evaluation seems to be a good way of identifying subclinical dysfunction of the middle ear and/or cochlea, problems which cannot be detected using traditional audiological assessments. Indeed, it seems that the use of TEOAEs based on an appropriate protocol and criteria set can be an efficient tool in school-age hearing screening [29,47].

The DPOAE responses were found to be significantly different at all frequencies tested, with responses for the control group more robust than those in the study group. It has already been found that a history of OME has the effect of lowering DPOAE amplitudes [14,17,21,24,25]. This impairment can cause minor subclinical dysfunction, but in the long term it can lead to irreversible damage to the middle ear or cochlea [17,21,24,25]. Duplessis and Fothergill [48] attributed reduced DPOAE amplitudes to the middle ear, so that there was a reduction in amplification by the middle ear. One study of DPOAE growth curves in individuals with middle ear alterations [16] found that DPOAEs could effectively identify changes in individuals with OME. In the present study, we observed a statistically significant difference in SNR values at all frequencies (except at 2542 Hz), whereas a similar study [16] found reduced DPOAE amplitudes at 2002, 3174, and 4004 Hz. Another study [49] also found DPOAEs to be sensitive measures of subclinical dysfunction. However, more work is still needed to clarify to what extent the reduction in TEOAE and DPOAE amplitudes interferes with higher-order auditory tasks. At the moment, it is suggested that lateral asymmetries may result from differences in the strength of efferent inhibition delivered to each cochlea. Another possibility is that lateral asymmetry in the efferent system may be related to the well-known cortical asymmetries that are believed to underline speech perception, speech production, and other hearing abilities [50]. It is already known that episodes of OME in early infancy can have a negative impact on learning and communication [51,52,53,54,55].

The 2016 guidelines on OME suggest that children with the condition may develop structural changes in the tympanic membrane, hearing loss, and delayed speech and language [35]. OME may lead to difficulties in speech and reading, delayed responses to auditory stimuli, limited vocabulary, and inattention, all of which may have an impact on the child’s school performance. In addition, the auditory deprivation caused by OME during critical periods in a child’s central auditory nervous system development can lead to auditory processing disorder. If auditory messages become distorted, there can be failures in the coding and organization of auditory processing [35,38,40,51,52,53,54,55].

For all these reasons, children with a history of OME should be regularly monitored, even if they have auditory thresholds within normal limits. Any absence of stimulation and/or changes in everyday sound stimuli run the risk of permanently affecting the central auditory nervous system.

## 5. Limitations and Future Research

In the present study, there was a predominance of females in both groups. Further studies should include equal numbers of males and females because females tend to have better OAE responses. Another issue for future studies might be to look more closely at the condition of the tympanic membrane, ascertaining possible fibrosis of the membrane due to recurrent OME, since this type of fibrosis might interfere in the transmission of OAEs.

## 6. Conclusions

Children with a history of recurrent bilateral otitis media who had submitted to surgery for insertion of bilateral ventilation tubes in the first years of life had reduced amplitudes of otoacoustic emissions compared with typical children. A history of repeated otitis media was found to interfere with the generation and transmission of TEOAEs and DPOAEs. Thus, we have found that TEOAE and DPOAEs tests are sensitive in identifying changes in patients with a history of otitis media.

## Figures and Tables

**Table 1:** Mean hearing thresholds in the right and left ears between the control and experimental groups.

	250 Hz	500 Hz	1000 Hz	2000 Hz	3000 Hz	4000 Hz	6000 Hz	8000 Hz
RE-CG	8 dB	7.5 dB	6.5 dB	6 dB	4.5 dB	5.5 dB	12.5 dB	8.5 dB
RE-EG	8.3 dB	7.2 dB	5.5 dB	5 dB	4.4 dB	5 dB	12.2 dB	7.2 dB
*p*-value	0.589	0.200	0.361	0.687	0.358	0.324	0.950	0.198
LE-CG	8 dB	7 dB	5 dB	7.5 dB	4 dB	7 dB	8.8 dB	6.5 dB
LE-EG	8.8 dB	6.1 dB	4.4 dB	7 dB	5 dB	5 dB	10 dB	5 dB
*p*-value	0.892	0.301	0.486	0.154	0.909	0.150	0.926	0.672

Legend: RE—Right Ear; LE—Left Ear; CG—Control Group; EG—Experimental Group. *p* ≤ 0.05.

**Table 2:** Presence or absence of TEOAEs and DPOAEs between groups.

		TEOAE				DPOAE			
		Present		Absent		Present		Absent	
		%	n	%	n	%	n	%	n
**CG**	RE LE	100 100	50 50	- -	- -	100 100	50 50	- -	-
**EC**	RE LE	82 84	41 42	18 16	9 8	92 90	46 45	8 10	4 5

Legend: TEOAE—Transient Evoked Otoacoustic Emission; DPOAE—Distortion-Product Otoacoustic 190 Emission; RE—Right Ear; LE—Left Ear; CG—Control Group; EG—Experimental Group.

**Table 3:** Comparison of TEOAE (1.2–3.4 kHz) results in terms of group, age, and gender as factors.

		Gender *Age		Gender *Group		Age *Group		Gender *Age *Group	
		*p*-Value		*p*-Value		*p*-Value		*p*-Value	
		RE	LE	RE	LE	RE	LE	RE	LE
1000 Hz	TE	0.661	0.318	0.615	0.161	0.219	0.351	0.993	0.873
	NF	0.63	0.712	0.631	0.095	0.34	0.806	0.765	0.300
	SNR	0.884	0.429	0.826	0.979	0.489	0.403	0.839	0.23
1500 Hz	TE	0.648	0.742	0.414	0.182	0.802	0.602	0.882	0.673
	NF	0.593	0.527	0.240	0.219	0.713	0.463	0.165	0.539
	SNR	0.842	0.858	0.841	0.669	0.954	0.228	0.419	0.953
2000 Hz	TE	0.915	0.804	0.421	0.330	0.660	0.554	0.741	0.745
	NF	0.627	0.733	0.952	0.282	0.629	0.675	0.747	0.359
	SNR	0.733	0.630	0.282	0.688	0.675	0.365	0.359	0.831
3000 Hz	TE	0.331	0.854	0.924	0.293	0.568	0.29	0.073	0.243
	NF	0.068	0.492	0.894	0.188	0.523	0.273	0.101	0.599
	SNR	0.913	0.975	0.878	0.476	0.454	0.683	0.313	0.266
4000 Hz	TE	0.918	0.218	0.108	0.818	0.776	0.663	0.617	0.831
	NF	0.142	0.546	0.612	0.833	0.175	0.064	0.039	0.210
	SNR	0.147	0.352	0.134	0.937	0.271	0.2	0.11	0.138
1.2–3.4 kHz	TE	0.696	0.682	0.847	0.159	0.956	0.939	0.361	0.987
	NF	0.805	0.69	0.795	0.157	0.259	0.356	0.102	0.405
	SNR	0.745	0.85	0.932	0.537	0.493	0.545	0.86	0.542

**Legend:** RE—right ear; LE—left ear; TE—transient emission; NF—noise floor; SNR—transient emission less noise floor. *p* ≤ 0.05.

**Table 4:** Results of TEOAE frequencies between RE (*n* = 41) and LE (*n* = 42).

		CG				EC				*p*-Value	
		Mean		SD		Mean		SD			
		RE	LE	RE	LE	RE	LE	RE	LE	RE	LE
**1000 Hz**	TE	2.1	2.2	5.8	5.42	−0.19	−1.8	4.7	4.9	0.000	0.000 *
	NF	−5.5	−6.2	3.2	4.15	−6.3	−5.2	3.5	4.6	0.248	0.289
	SNR	7.6	8.4	4.8	4.6	4.4	3.4	4.6	4.2	0.001 *	0.000 *
**1500 Hz**	TE	7.6	7.1	5.9	5.3	2.3	2.3	5.6	5.3	0.000 *	0.000 *
	NF	−4.5	−4.6	3.2	4.2	−6.1	−5.7	3.4	4.0	0.027 *	0.216
	SNR	12.1	11.7	4.5	5.1	8.4	8.1	4.5	4.4	0.000 *	0.000 *
**2000 Hz**	TE	4.3	4.7	4.8	3.7	2.1	2.2	4.8	5.1	0.036 *	0.009 *
	NF	−6.1	−5.8	2.2	2.2	−6.1	−5.5	2.2	2.8	0.775	0.618
	SNR	10.4	10.5	4.5	3.7	8.2	7.7	4.1	4.3	0.014 *	0.001 *
**3000 Hz**	TE	5.2	4.6	5.61	5.1	1.8	1.8	4.9	5.6	0.003 *	0.014 *
	NF	−4.9	−5.8	3.3	1.5	−5.7	−5.6	1.7	1.8	0.192	0.419
	SNR	10.1	10.4	5.7	4.5	7.5	7.4	4.4	5.0	0.023 *	0.003 *
**4000 Hz**	TE	3.4	3.2	4.9	4.1	−2.3	−1.4	5.6	6.1	0.000 *	**0.000**
	NF	5.8	−6.4	4.6	3.8	−7.2	−7.2	4.7	4.4	0.225	0.320
	SNR	9.3	9.6	4.3	4.4	4.9	5.8	3.7	4.0	0.000 *	0.000 *
**1.2–3.4 kHz**	TE	11.8	11.1	4.7	3.9	7.4	7.5	4.7	4.7	<0.000 *	<0.000 *
	NF	−0.3	−0.1	2.3	2.8	−0.8	−0.5	2.3	2.6	0.349	0.523
	SNR	12.1	11.3	3.9	3.4	8.3	8.0	3.9	3.7	<0.000 *	<0.000 *

**Legend:** Hz—hertz; SD—standard deviation; RE—right ear; LE—left ear; TE—transient emission; NF—noise floor; SNR—transient emission less noise floor, Bold numbers—*p* ≤ 0.05 *.

**Table 5:** Comparison of DPOAE (1818–7206 Hz) results in terms of group, age, and gender.

		Gender *Age		Gender *Group		Age *Group		Gender *Age *Group	
		*p*-Value		*p*-Value		*p*-Value		*p*-Value	
		RE	LE	RE	LE	RE	LE	RE	LE
7206 Hz	DP	0.246	0.097	0.682	0.469	0.971	0.562	0.506	0.903
	NF	0.216	0.299	0.249	0.300	0.49	0.738	0.422	0.723
	SNR	0.627	0.261	0.860	0.824	0.764	0.687	0.816	0.782
5083 Hz	DP	0.481	0.565	0.828	0.913	0.725	0.775	0.648	0.616
	NF	0.501	0.584	0.8311	0.615	0.809	0.356	0.650	0.379
	SNR	0.514	0.877	0.766	0.842	0.103	0.767	0.909	0.923
3616 Hz	DP	0.785	0.863	0.964	0.790	0.978	0.411	0.452	0.457
	NF	0.444	0.496	0.305	0.159	0.083	0.565	0.544	0.631
	SNR	0.784	0.571	0.501	0.482	0.288	0.323	0.853	0.384
2542 Hz	DP	0.742	0.126	0.918	0.872	0.846	0.973	0.257	0.669
	NF	0.843	0.595	0.646	0.989	0.462	0.961	0.34	0.289
	SNR	0.909	0.477	0.802	0.914	0.498	0.950	0.842	0.601
1818 Hz	DP	0.893	0.927	0.185	0.070	0.320	0.547	0.651	0.696
	NF	0.681	0.978	0.843	0.368	0.949	0.875	0.542	0.224
	SNR	0.697	0.916	0.379	0.530	0.402	0.736	0.934	0.414

**Legend:** RE—right ear; LE—left ear; DP—distortion production emission; NF—noise floor; SNR—signal to noise ratio, Bold numbers—*p* ≤ 0.05 *.

**Table 6:** Results of DPOAE (1818–7206 Hz) between RE (*n* = 46) and LE (*n* = 45).

−		CG				EG					
		Mean		SD		Mean		SD		*p*-Value	
Frequency GM (HZ)		RE	LE	RE	LE	RE	LE	RE	LE	RE	LE
7206	DP	2.6	2.5	9.8	9.1	−6.1	−6.7	10.7	11.1	<0.001 *	<0.000 *
	NF	17.0	−16.1	4.5	3.7	−18.0	−18.4	6.0	5.7	0.351	0.017 *
	SNR	19.6	18.7	10.3	8.9	11.9	11.6	11.5	11.9	<0.001 *	0.001 *
5083	DP	3.3	5.2	7.6	5.9	−4.4	−3.4	11.0	12.2	<0.000 *	<0.000 *
	NF	17.5	−16.3	4.7	5.5	−20.2	−17.9	8.6	8.2	0.029 *	0.169
	SNR	20.8	21.6	8.7	8.0	15.8	14.4	14.4	15.0	0.073	0.008 *
3616	DP	4.3	3.4	5.5	4.8	−2.0	0.1	10.1	8.1	<0.000 *	0.031 *
	NF	15.7	−15.7	4.4	4.4	−15.7	−16.0	8.5	6.5	0.870	0.657
	SNR	20.1	19.1	6.4	6.6	13.7	16.2	14.2	10.6	0.011 *	0.173
2542	DP	6.3	6.2	6.1	5.6	2.8	1.6	12.3	7.7	0.145	0.002 *
	NF	12.1	−12.5	6.4	5.6	−12.7	−13.4	10.5	8.4	0.639	0.512
	SNR	18.4	18.7	6.8	6.5	15.5	15.0	16.2	11.2	0.423	0.078
1818	DP	9.8	10.6	7.4	5.1	4.2	5.0	7.6	7.2	<0.001 *	<0.000 *
	NF	−7.8	−7.6	5.2	5.1	−9.2	−8.3	7.7	9.0	0.299	0.471
	SNR	17.7	18.2	8.6	5.7	13.4	13.4	10.8	9.3	0.062	0.005 *

**Legend:** SD—standard deviation; GM—geometric mean; RE—right ear; LE—left ear; DP—distortion production emission; NF—noise floor; SNR—signal to noise ratio; *n*—number of ears, Bold numbers—*p* ≤ 0.05 *.
